# Non-tuberculous Mycobacterial Infection of the Musculoskeletal System Detected at Two Tertiary Medical Centres in Henan, China, 2016–2020

**DOI:** 10.3389/fmicb.2021.791918

**Published:** 2021-12-16

**Authors:** Qiong Ma, Rende Chen, Enhui Yang, Youhua Yuan, Yongfu Tian, Yongguang Han, Shanmei Wang, Baoya Wang, Wenjuan Yan, Qi Zhang, Nan Jing, Bing Ma, Zhen Wang, Yi Li, Yongjun Li

**Affiliations:** ^1^Henan Provincial People’s Hospital, People’s Hospital of Zhengzhou University, Henan University People’s Hospital, Zhengzhou, China; ^2^Luoyang Orthopedic Hospital of Henan Province, Orthopedic Hospital of Henan Province, Zhengzhou, China; ^3^School of Pharmacy, Henan University of Chinese Medicine, Zhengzhou, China

**Keywords:** musculoskeletal infection, non-tuberculous mycobacterium, diagnosis, surgical treatment, antimycobacterial drug, outcomes

## Abstract

Non-tuberculous mycobacterial (NTM) infection of the musculoskeletal system is rare but poses a grave threat to public health. These infections yield non-specific symptoms that remain undetected until the development of the later stages of the disease. In this study, we performed a retrospective review of 25 cases of musculoskeletal NTM infection at two tertiary medical centres over a 5-year period to determine the clinical features and improve the current clinical diagnosis and treatment. The most common mycobacterial species detected were *Mycobacterium fortuitum* in eleven patients, *Mycobacterium abscessus* in eight patients, *Mycobacterium houstonense* in three patients, *Mycobacterium avium* in two patients, and *Mycobacterium smegmatis* in one patient. The sites of infection included the limbs and joints, most commonly the knee (ten patients) and foot (six patients). The median duration from the onset of symptoms to diagnosis was 2.5 months (0.8–13.5 months). Deep sinus tracts extending to the surgical site were observed in 60% of the patients (15/25), and granulomatous inflammation and granulomatous inflammation with necrosis occurred in 60% of the patients (15/25). All patients underwent surgical treatment for infection control, and all patients, except one, received antimycobacterial therapy based on drug sensitivity assays. The median duration of the antimicrobial chemotherapy was 5 months (range: 3–20 months). At the final follow-up, 24 patients presented with absence of recurrence and one patient succumbed owing to heart failure after debridement. Our findings highlight the importance of vigilance and improvements in the diagnostic methods for musculoskeletal NTM infection. Aggressive surgical treatment and antimycobacterial drug treatment can help achieve satisfactory results.

## Introduction

All *Mycobacterium* species, except the *Mycobacterium tuberculosis* complex and *Mycobacterium leprae*, are referred to as non-tuberculous mycobacterial (NTM) species. These species are ubiquitous in nature (Daley et al., 2020; Johansen et al., 2020). More than 120 NTM species have been reported to cause human infections, including opportunistic pulmonary and extrapulmonary infections, among which pulmonary NTM infection is the most commonly documented infection type (Cassidy et al., 2009; Haworth et al., 2017; Daley et al., 2020; Johansen et al., 2020). The incidence and the prevalence of NTM infections vary significantly among studies available in the literature (Smith et al., 2017; Shih et al., 2018; Furuuchi et al., 2019; Winthrop et al., 2020). Epidemiological studies of NTM infections cannot be easily conducted, and the collection of precise data from different countries and regions is challenging because public health reporting is not necessary in most countries. However, according to available data, the incidence and prevalence of NTM infections continue to increase in certain countries. A study conducted in Japan showed that the incidence of NTM infections increased from 4.6 per 100,000 in 2001 to 10.1 per 100,000 in 2009, and the prevalence of NTM infections in North Korea increased from 9.4 per 100,000 in 2009 to 36.1 per 100,000 in 2016 (Yoon et al., 2017; Furuuchi et al., 2019). The trend may be explained by improvements in laboratory culture methods, increased clinical awareness, increased number of immunosuppressed individuals in the population, long-term usage of antimicrobial drugs and immunosuppressive drugs, and increased environmental exposure (e.g., widespread use of water heaters and exposure shower aerosols) (Bryant et al., 2013, 2016; Haworth et al., 2017; Lee et al., 2019; Daley et al., 2020; Gopalaswamy et al., 2020). Currently, the management of extrapulmonary infection caused by NTM species is based on the information available in case reports and series. Skin and soft tissue infections are the most commonly occurring extrapulmonary NTM infections, while musculoskeletal infections with either fast- or slow-growing mycobacteria are less common (Goldstein et al., 2019).

Previous case reports and case series published on musculoskeletal NTM infections have focused on the risk factors, clinical manifestations, diagnosis, treatments, and outcomes (Garcia et al., 2013; Lin et al., 2014; Park et al., 2014; Nguyen et al., 2015; Wood et al., 2015; Kuntz et al., 2016; Gundavda et al., 2017; Yoon et al., 2017; Holt and Kasperbauer, 2018; Goldstein et al., 2019; Hsueh et al., 2019; Napaumpaiporn and Katchamart, 2019; Kwan and Tupler, 2021). In a series of 28 cases reported in Thailand (Napaumpaiporn and Katchamart, 2019), 25% of the cases presented with previous musculoskeletal trauma, 18% presented with prior subjection to bone and joint surgery, 14% presented with prosthetic joint replacement, and 11% presented with HIV infection. While most patients underwent surgery (82%), and 18% received only antibiotic treatment, complete recovery was only observed in 46% of the patients. Improvement with residual disability and deformities was observed in 29% of the patients, and 3.6% succumbed to the disease. In another series, all 14 cases of non-spinal musculoskeletal NTM infections reported in a tertiary referral centre in the United States presented with treatment regimens of multiple antimicrobial agents along with aggressive surgical treatment; 13 of the 14 patients were ultimately cured (Goldstein et al., 2019). This study highlighted the importance of careful monitoring for musculoskeletal NTM infections in immunosuppressed patients or those with a history of musculoskeletal surgery. In a series of 29 cases reported in South Korea, all patients were subjected to surgical intervention in addition to the prescription of antimicrobial drugs (Park et al., 2014). Twenty patients among such patients received specific medications for NTM infections, and nine received conventional antimicrobial therapy. At the follow-up, 22 of the 29 patients were found to be cured.

In all cases mentioned above, the infection was acquired because of penetrating trauma, such as injury caused by acupuncture or intraarticular injections, in which contamination occurred during surgical procedures. Infection was also prevalent among immunocompromised individuals. The diagnosis of musculoskeletal NTM infection is often delayed by the indolent course of infection and the lack of availability of appropriate diagnostic tests. Once diagnosed, the infection can be effectively treated using a combination of debridement and anti-NTM infection chemotherapy. Therefore, there is a need for acquisition of more clinical data that can be used to provide guidance to the treatment strategies for patients with severe musculoskeletal NTM infections. In this study, we aimed to determine the clinical features and treatment outcomes of musculoskeletal NTM infections in China.

## Materials and Methods

### Study Design

This was a retrospective study involving patients with musculoskeletal NTM infections treated at Henan Provincial People’s Hospital (*n* = 6) and Henan Provincial Orthopaedic Hospital (*n* = 19) during the period 2016–2020. The two hospitals are tertiary medical centres. Additional data for each positive culture included patient characteristics, clinical manifestations, isolation of NTM species from the samples collected, source of the isolate, investigations, treatments, and outcomes. Specimens for culture and metagenomic next-generation sequencing (NGS) were obtained at the same time from the infected site during surgery under sterile conditions. The specimens were cultured under standard mycobacterial conditions at 30°C and 35°C in liquid BACTEC Mycobacteria Growth Indicator Tubes (MGIT; Becton Dickinson, Sparks, MD, United States) and a solid 3% Ogawa medium (Baso Diagnostics, Inc., Zhuhai, China), respectively. All isolates were identified at the species or complex level *via* matrix-assisted laser desorption/ionisation-time of flight mass spectrometry. All NTM species were confirmed by Beijing Ruiboxing Co., Ltd. based on *16S rRNA*, *ropB, gyrB, SecA1*, or *hsp65* gene sequencing experiments. Susceptibility testing was performed on rapidly growing mycobacterial samples using a standard broth microdilution method on Sensititre Susceptibility plates (Trek Diagnosis Systems/Biocentric, Bandol, France). Slow-growing mycobacteria were tested using Sensititre MAISLOW plates (Biocentric). If musculoskeletal NTM infection was confirmed, clinical treatment was based on the guidelines for diagnosis and treatment of non-tuberculous mycobacteria diseases (Tang et al., 2020) and The Sanford Guide to Antimicrobial Therapy (David et al., 2020). After the sensitivity of musculoskeletal NTM infection had been determined, the initial chemotherapy was adjusted.

Metagenomic NGS analysis of tissue samples was performed by Vision Medical Co., Ltd. (Zhuhai, China).

### Case Definition and Data Collection

A case of musculoskeletal NTM was defined as a culture-confirmed musculoskeletal NTM infection involving biopsy of tissue or puncture during surgery, with treatment information based on a registry of musculoskeletal NTM infections at the two hospitals. Clinical features, such as swelling, pain, tenderness, or redness, indicated the presence of chronic infection; additionally, investigations were based on the erythrocyte sedimentation rate, C-reactive protein levels, total leucocyte counts, white blood cell differentiated counts, histopathological analyses, and imaging. Data related to the time to symptom onset, total duration of therapy, and time from last infection control surgery to cessation of antimicrobial therapy were collected from patients with different NTM strains and different immune states. Criteria for an immunosuppressed state were based on the definition of Bonten et al. (2015).

### Statistical Analysis

Demographic characteristics and data for concurrent conditions, age, sex, species, laboratory values, surgical intervention, antimycobacterial chemotherapy, and outcomes were evaluated. For quantitative data, descriptive statistics have been depicted as median values. Qualitative data have been presented in the form of numbers and percentages (%). Figures were created with the Prism 8.0 (GraphPad) software. Other statistical analyses were performed with the SPSS 20.0 software (IBM Corporation, Armonk, NY, United States). Two-tailed *P* values < 0.05 were considered as statistically significant.

## Results

### Patient Characteristics and Clinical Manifestations

Over the 5-year study period, the incidence of NTM infection increased. NTM infection was diagnosed in two patients in 2016, two patients in 2017, four patients in 2018, eight patients in 2019, and nine patients in 2020. The mean age ± standard deviation (SD) was 54.14 ± 16.05 years, and 14 of these patients were male. Ten patients presented with underlying diseases, and three were immunosuppressed patients. The common predisposition factors for musculoskeletal NTM infections included previous history of musculoskeletal trauma (*n* = 12), such as a fall, exposure to a foreign body, lacerations, prior bone and joint surgery (*n* = 3), and percutaneous infection (*n* = 8), including those due to acupuncture, sodium hyaluronate administration, and steroid injections, or intravenous injections. The most frequently involved sites comprised the knee (*n* = 10) and foot (*n* = 6), followed by the ankle (*n* = 3), thigh (*n* = 2), lumbar spine (*n* = 1), hip (*n* = 1), forearm (*n* = 1), tibia (*n* = 1), and femur (*n* = 1).

**FIGURE 1:**
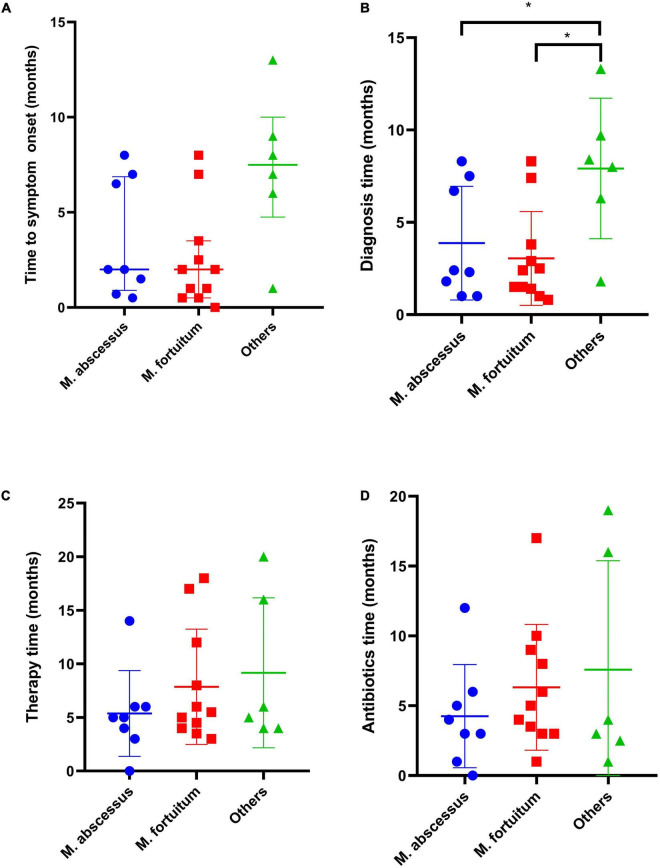
Comparison of clinical manifestations and duration of therapy for *M. abscessus* (*n* = 8), *M. fortuitum* (*n* = 11), and others (*n* = 6). **(A)** Time to symptom onset, **(B)** Time from symptom onset to diagnosis, **(C)** Total duration of therapy, months, and **(D)** Time from last infection control surgery to cessation of antimicrobial therapy, months. *p < 0.05.

**FIGURE 2:**
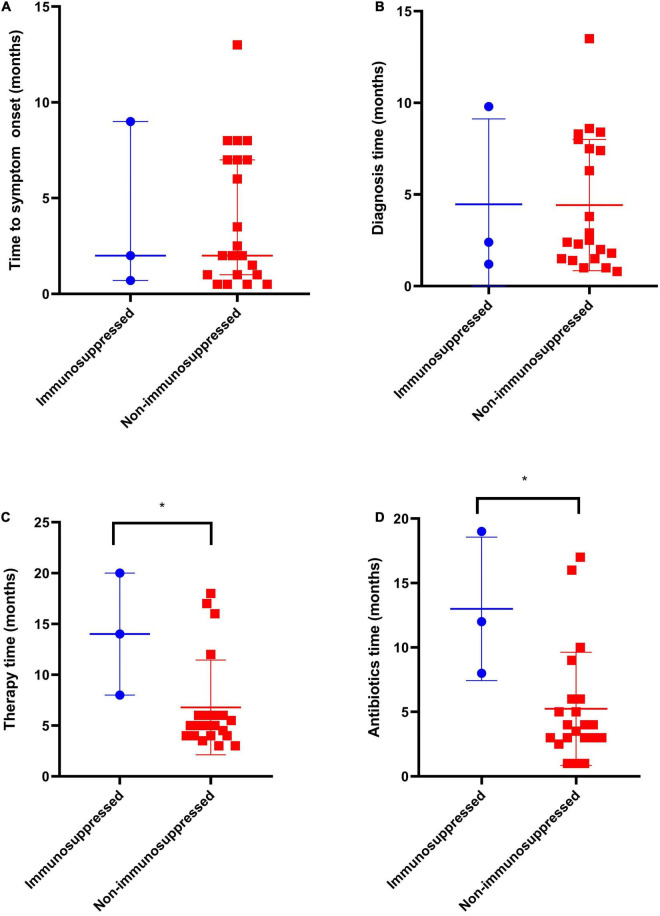
Comparison of clinical manifestations and duration of therapy of immunosuppressed (*n* = 3) and non-immunosuppressed patients (*n* = 21). **(A)** Time to symptom onset, **(B)** Time from symptom onset to diagnosis, **(C)** Total duration of therapy, months, and **(D)** Time from last infection control surgery to cessation of antimicrobial therapy, months. *p < 0.05.

Twenty-three patients were referred from lower-level hospitals; two patients (cases 9 and 12) were admitted to the Henan Provincial Orthopaedic Hospital with multiple injuries. The most common clinical manifestations in these patients were osteomyelitis (*n* = 12), septic arthritis (*n* = 6), infected total knee arthroplasty (*n* = 1), soft tissue infection (*n* = 3), wound infection (*n* = 2), Synovitis (*n* = 1), and lumbar spinal abscess (*n* = 1). The main complaints included purulent discharging sinuses, unhealed wounds/bleeding, pain, swelling, redness, warmth at the affected site, tenderness, restricted/painful joint movement, and fever. The median duration of the onset of symptoms was 2 months (range, 0.5–13 months), and the median duration between the onset of symptoms and diagnosis was 2.5 months (range, 0.8–13.5 months) (Table 1). The time from symptom onset to diagnosis for *M. abscessus* and *Mycobacterium fortuitum* was shorter as compared to that for other NTM strains, while there was no significant difference in the time to symptom onset of different strains (Figure 1). There was no significant difference regarding the time to symptom onset and the time from symptom onset to diagnosis between immunosuppressed and non-immunosuppressed patients (Figure 2).

**TABLE 1:** Clinical manifestations and correlated findings of 25 patients with musculoskeletal non-tuberculous mycobacterial infections in Henan, China.

Case no.	Age (y)/sex	Inciting or predisposing factor(s)	Initial presentation	Time to symptom onset (months)	Reason for which care was sought	Metagenomic NGS results	Histopathological	AFB	MR imaging	Time from symptom onset to diagnosis (months)
1	49/M	Trauma	Open fracture	7	Osteomyelitis	ND	Granulomatous inflammation	Negative	Soft tissue swelling, osteopenia, sinus tracts	8
2	73/M	TKA	Infected TKA	0.7	Infected TKA	ND	Granulomatous inflammation	Negative	Joint effusion and soft tissue swelling, sinus tracts	1.2
3	77/F	Wrist surgery	Tenosynovitis	6.5	Soft tissue infection	ND	Granulomatous inflammation with necrosis	Negative	None	6.7
4	46/M	Laceration	Osteoarthritis	13	Osteomyelitis	ND	Granulomatous inflammation with necrosis	1 to 9/10 HPF	Soft tissue swelling, osteopenia, osseous erosions, sinus tracts	13.5
5	61/M	Fall	Open fracture	8	Osteomyelitis	ND	Acute/chronic inflammation	Negative	Joint effusion and soft tissue swelling, osteopenia, sinus tracts	8.6
6	46/M	Sodium hyaluronate injection	Arthritis	2	Septic arthritis	ND	Granulomatous inflammation	Negative	Joint effusion and soft tissue swelling sinus tracts	2.4
7	63/F	Acupuncture	Arthritis	6	Osteomyelitis	ND	Granulomatous inflammation	Negative	Joint effusion and soft tissue swelling, osteopenia	6.3
8	44/M	Trauma	Achilles’ tendon rupture	0.5	Osteomyelitis	ND	Granulomatous inflammation with necrosis	1 to 2/300 HPF	Soft tissue swelling, sinus tracts	1
9	28/F	Multiple traumas	Multiple traumas	0.5	Draining wound at stump	ND	Acute/chronic inflammation	Negative	Soft tissue swelling, osteopenia, sinus tracts	1.5
10	51/M	Acupuncture, sodium hyaluronate injection	Arthritis	2	Synovitis, septic arthritis	ND	Granulomatous inflammation	Negative	Soft tissue swelling, osteopenia	2.3
11	56/F	Acupuncture	Synovitis	2.5	Septic arthritis	*M. fortuitum*	None	None	Joint effusion and soft tissue swelling	2.9
12	36/M	Trauma	Open fracture	0.5	Septic arthritis	*M. abscessus*	Acute/chronic inflammation	Negative	Soft tissue swelling, osteopenia	1
13	54/M	Fall	Open fracture	8	Osteomyelitis	*M. houstonense*	Granulomatous inflammation	Negative	Joint effusion and soft tissue swelling osteopenia, sinus tracts	8.4
14	74/F	Acupuncture	Arthritis	3.5	Septic arthritis	*M. fortuitum*	Granulomatous inflammation	Negative	Joint effusion and soft tissue swelling, sinus tracts	3.8
15	46/F	Steroid injection	Osteoarthritis	9	Osteomyelitis	*M. avium*	Granulomatous inflammation with necrosis	1 to 9/10 HPF	Soft tissue swelling, osteopenia, osseous erosions	9.8
16	56/F	Fracture internal fixation	Fracture	1.5	Osteomyelitis	ND	Granulomatous inflammation	Negative	Soft tissue swelling, osteopenia, sinus tracts	1.8
17	30/M	Foreign body	Blunt trauma	2	Osteomyelitis	*M. abscessus*	Granulomatous inflammation with necrosis	1 to 2/300 HPF	Joint effusion and soft tissue swelling, osteopenia, sinus tracts	2.4
18	75/F	None	Lumbar disc protrusion	7	Lumbar vertebral body destruction with abscess	*M. abscessus*	Granulomatous inflammation with abscess	1 to 9/10 HPF	Signal abnormality in second and third lumbar vertebral bodies with abscess formation	7.5
19	27/M	Trauma	Open fracture	1	Osteomyelitis	ND	Acute/chronic inflammation	Negative	Soft tissue swelling, osteopenia, osseous erosions, sinus tracts,	1.5
20	34/M	Steroid injection hip with buried suture	Lumbar disc protrusion	1	Soft tissue infection	*M. fortuitum*	Acute/chronic inflammation	Negative	None	1.4
21	63/M	Foreign body	Open fracture	0.5	Draining wound at stump	ND	Acute/chronic inflammation	Negative	Soft tissue swelling, osteopenia	0.8
22	28/F	Foreign body	Blunt trauma	1	Soft tissue infection	*M. avium*	Acute/chronic inflammation	Negative	Soft tissue swelling, sinus tracts	2
23	25/M	Trauma	Open fracture	2	Osteomyelitis	*M. fortuitum*	Acute/chronic inflammation	Negative	Joint effusion and soft tissue swelling, sinus tracts	2.5
24	54/F	None	Thigh abscesses	8	Osteomyelitis	*M. fortuitum*	Granulomatous inflammation with necrosis	Negative	Soft tissue swelling sinus tracts	8.3
25	36/F	Sodium hyaluronate injection	Arthritis	7	Septic arthritis	ND	Acute/chronic inflammation	Negative	Soft tissue swelling, osteopenia	7.4

*AFB, acid-fast bacilli; HPF, high power field. ND, not detected; TKA, total knee arthroplasty.*

### Diagnosis

All patients presented with positive mycobacterial cultures at the time of initial diagnosis at the two tertiary hospitals. The specimen types for pathogenic diagnosis were synovial biopsy (*n* = 22), aspirate specimen (*n* = 1), synovial fluid (*n* = 2), and abscess specimen (*n* = 1), and two or three tissue samples were collected from each patient for culture. Twenty-two patients were found to be infected with a rapidly growing NTM species, including the presence of *M. fortuitum* in twelve patients, *M. abscessus* in seven patients, *M. houstonense* in two patients, and *M. smegmatis* in one patient. The remaining two patients were infected with *M. avium*, a slow-growing species. Since 2019, 10 tissue specimens have been analysed using metagenomic NGS, and the NTM species detection results were consistent with the culture results (Table 1).

The median erythrocyte sedimentation rate (ESR) was estimated to be above the upper limit of normal (ULN) in 17 patients (68%) at arrival (ULN 15 mm/h in men and 20 mm/h in women). C-reactive protein levels were found to be elevated in 13 patients (52%) (ULN 10.0 mg/L). The blood leucocyte count was found to be elevated in two patients (8%) (ULN 10.1 × 10^4^ cells/μL). All three laboratory tests were within reference limits in four patients. However, in one patient (case 3), three laboratory tests were above the upper limit of the reference range.

**FIGURE 3:**
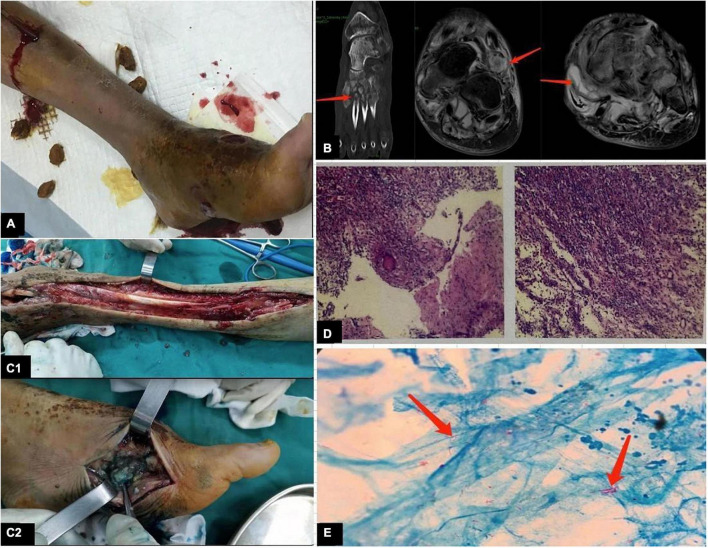
(Case 4) **(A)** Clinical photographs show markedly swollen left foot, discharging sinus in the dorsum and inner side of the foot, and a skin drainage wound at the proximal end of the leg. **(B)** Magnetic resonance imaging demonstrates bone erosion, bone destruction, and decreased bone density in the metatarsal bone; swelling of the surrounding soft tissue structures; and sinusoidal shadows around the medial foot. **(C1)** The medial side of the foot reveals the dorsalis and plantar proliferative inflammatory tissues. **(C2)** Intraoperative photographs show local bone destruction with more inflammatory granulation tissue extending proximal to the tendon. **(D)** Histopathology microphotograph shows granulomatous inflammation with necrosis. **(E)** Intraoperative tissue samples with the presence of acid-fast bacilli (arrow).

Magnetic resonance (MR) imaging was performed for 23 patients but was non-specific for musculoskeletal NTM infection. The findings included soft tissue swelling (*n* = 22), joint effusion (*n* = 10), sinus tracts (*n* = 13), osteopenia (*n* = 14), and osseous erosions (*n* = 4). MR imaging of a patient (case 18) showed signal abnormalities of the second and third lumbar vertebral bodies with abscess formation. Histopathologic examinations of 24 patients were performed. A spectrum of inflammatory changes was observed, including acute/chronic inflammation in nine patients, granulomatous inflammation in nine patients, and granulomatous inflammation with necrosis in six patients. Acid-fast-positive bacilli were detected in the surgical specimens derived from five patients. Preoperative and intraoperative clinical photographs; MR images; and results of histopathologic examinations and acid-fast bacilli detection of case 4 are shown in the Figure 3.

### Treatment and Outcomes

All patients underwent surgical treatment for infection control, and specific antimicrobial therapy for NTM infections was administered to all but one patient. One patient with an infection caused by *M. abscessus* (case 3) succumbed because of heart failure after debridement. The remaining 24 patients presented with complete cure after follow-up. Ten patients presented with ≥1 debridement before seeking treatment at the tertiary medical centres, and the infection was evidently not controlled (Table 2).

**TABLE 2:** Surgical and antimicrobial treatment of 25 patients with musculoskeletal non-tuberculous mycobacterial infections, Henan, China.

Case no.	Involved site	Species	Previous surgeries	Surgeries for control of infection at two tertiary medical centres	Oral therapy (duration, months)	Intravenous therapy (duration, months)	Total duration of therapy, months	Time from last infection control surgery to cessation of antimicrobial therapy, months	Outcome
1	Left tibia	*M. smegmatis*	Application external fixative, debridement	Debridement four times	Clarithromycin (4)	Cefoxitin + amikacin (2)	4	1	Cured
2	Right knee	*M. abscessus*	Resection arthroplasty	Debridement and vancomycin cement spacer, revision TKA, intramedullary nail with gentamycin cement insertion	Clarithromycin, linezolid (14)	Cefoxitin + amikacin + imipenem (6)	14	12	Cured
3	Right forearm	*M. abscessus*	Wrist surgery	Debridement, excision of soft tissue masses	None	None	0	0	Dead
4	Left foot	*M. houstonense*	Debridement	Debridement two times	Clarithromycin + ethambutol (5)	Cefoxitin + amikacin (1)	5	3	Cured
5	Left ankle	*M. abscessus*	Application internal fixative	Debridement and tobramycin cement beads insertion	Clarithromycin + linezolid (3)	Cefoxitin + amikacin (2)	3	3	Cured
6	Right knee	*M. fortuitum*	Debridement	Exploration and debridement	Azithromycin + minocyline (8)	Amikacin + imipenem (3.5)	8	8	Cured
7	Left knee	*M. houstonense*	Debridement	Debridement two times	Clarithromycin (4)	Amikacin + imipenem + tigecycline (1)	4	2.5	Cured
8	Right foot	*M. fortuitum*	Achilles’ tendon repair surgery	Debridement	Clarithromycin + minocyline (3.5)	Amikacin + imipenem (1.5)	3.5	3.5	Cured
9	Right femur, Right ankle	*M. fortuitum*	None	Right femur: application of external fixator, vancomycin bead implantation; left ankle: application internal fixative, irrigation, and debridement three times, internal fixative removal, pantalar arthrodesis, amikacin and meropenem cement insertion, VSD	Clarithromycin + ciprofloxacin + minocyline + sulfamethoxazole (18)	Amikacin (8)	18	10	Cured
10	Right knee	*M. abscessus*	None	Arthroscopy and synovectomy, debridement	Azithromycin (5)	Cefoxitin + amikacin (2)	5	5	Cured
11	Both knees	*M. fortuitum*	None	Abscess drainage	Ciprofloxacin + minocyline (3)	None	3	3	Cured
12	Left foot	*M. abscessus*	Application of external fixative	Excision of subcutaneous necrotic tissue, free skin flap transplantation, Debridement four times	Azithromycin + linezolid (6)	Amikacin (4)	6	1	Cured
13	Left knee	*M. houstonense*	Application of internal fixative	internal fixative removal, debridement two times	Clarithromycin (6)	Cefoxitin + amikacin (2)	6	4	Cured
14	Left knee	*M. fortuitum*	Debridement	Debridement and VSD	Clarithromycin + minocyline (4)	Amikacin + imipenem (1)	4	4	Cured
15	Right knee	*M. avium*	None	Debridement	Clarithromycin + ethambutol + rifampin (20)	Amikacin (8)	20	19	Cured
16	Right foot	*M. abscessus*	Application of internal fixative, explantation, debridement	Debridement, gentamycin, and vancomycin comment insertion	Azithromycin + linezolid (6)	Amikacin + imipenem (1)	6	6	Cured
17	Right foot	*M. abscessus*	None	Debridement five times, gentamycin bead implantation, VSD	Azithromycin + linezolid (5)	Cefoxitin + amikacin (2)	5	3	Cured
18	Lumber spine	*M. abscessus*	None	Debridement, amikacin cement insertion	Azithromycin + linezolid (4)	Cefoxitin + amikacin (1)	4	4	Cured
19	Right foot	*M. fortuitum*	Application of external fixative, debridement,	Debridement three times, gentamycin and meropenem cement insertion	Clarithromycin + sulfamethoxazole (12)	Amikacin + imipenem (3)	12	9	Cured
20	Right hip	*M. fortuitum*	None	Debridement	Clarithromycin + ciprofloxacin (17)	Amikacin (6)	17	17	Cured
21	Rightcankle	*M. fortuitum*	Application of external fixative, debridement	Debridement three times, VSD, pantalar arthrodesis, pedicled skin flap transplantation	Clarithromycin + ciprofloxacin (4.5)	Amikacin + tigecycline (2)	4.5	1	Cured
22	Right thigh	*M. avium*	Debridement	Debridement	Clarithromycin + ciprofloxacin (16)	Amikacin (5.5)	16	16	Cured
23	Right knee	*M. fortuitum*	Debridement, application internal fixative, VSD	Debridement, internal fixative removal, insertion of amikacin comment bead	Clarithromycin + minocyline (5.5)	Amikacin + imipenem (1)	5.5	3	Cured
24	Right thigh	*M. fortuitum*	Abscess drainage,	Debridement	Clarithromycin + ciprofloxacin (6)	Amikacin + imipenem (0.5)	6	6	Cured
25	Right knee	*M. fortuitum*	None	Debridement	Clarithromycin + ciprofloxacin (5)	Amikacin (1)	5	5	Cured

*VSD, vacuum sealing drainage.*

Surgical treatment at the two tertiary hospitals included aggressive debridement of infected bone and soft tissues as well as explanations of the infected hardware or internal fixation. For all but two patients (cases 1 and 9), NTM infection was confirmed in the first surgical specimen. Cases from five patients with *M. abscessus* (cases 2, 5, 16, 17, and 18) and three with *M. fortuitum* (cases 19 and 23) were treated extensively with antibacterial beads and bone cement during debridement and surgery. One patient with *M. fortuitum* (case 23) and one patient with *M. houstonense* (case 13) were subjected to the removal of internal fixation to control infection. The patient with *M. fortuitum* (case 9), who experienced multiple traumas, underwent seven procedures, including three debridements, femoral external fixator, internal ankle fixator, internal fixation removal with amikacin and meropenem cement, and subtalar arthrodesis for a period of 10 months. Two patients with *M. avium* (cases 15 and 22) underwent only one debridement operation, including an immunosuppressed patient (case 15; tested positive for acid-fast-positive bacilli), and received streptomycin-loaded and isoniazid-loaded cement during the operation. The patient with *M. abscessus* (case 3) was admitted in the hospital with an infection in the right forearm and succumbed because of heart failure following debridement and soft tissue excision.

All patients were subjected to treatment with combinatorial antimycobacterial drugs, determined based on antimicrobial drug sensitivities (Supplementary Table 1) and recommendations from the two tertiary medical centres. One (case 1) of two patients (cases 1 and 9) with open fractures caused by trauma suffered from multiple infections with *M. smegmatis* and *Klebsiella pneumoniae*, while the other patient (case 9) was simultaneously infected with *M. fortuitum*, *Pseudomonas aeruginosa*, and *Staphylococcus aureus*, and antimicrobial therapy was performed based on antibiotic sensitivities. During antimycobacterial chemotherapy treatment, few patients experienced adverse reactions to the drugs. Amikacin-related toxicity occurred in two patients. A patient (case 6) reported tinnitus after subjection to 3 months of parenteral amikacin treatment. One of the patients (case 9) experienced tinnitus 2 months after parenteral amikacin treatment and 1 month after being subjected to the implantation of amikacin beads, with severe gastrointestinal reactions occurring 4 months after the administration of ciprofloxacin. Treatment in one patient (case 2) was discontinued because of the development of leucopenia and thrombocytopaenia after 8 months of oral linezolid treatment. One of the patients (case 15) presented with a transient elevation of transaminase level due to the oral administration of rifampicin and then the patient recovered spontaneously. Seven patients (cases 4, 6, 13, 16, 17, 20, and 22) demonstrated temporary abnormalities in liver and kidney function indices and adverse reactions in the gastrointestinal tract, with recovery observed and documented after follow-up review.

The median duration of antimicrobial chemotherapy, except in one patient (case 3), was 5 months (range, 3–20 months). Among the 22 patients, excluding one patient (case 3) with rapidly growing NTM strains, the median duration was 5 months (range, 3–18 months). The treatment durations for the two patients with slow-growing NTM strains were 20 months and 16 months, respectively. All patients that received antimicrobial injections, as part of their treatment regimens, were treated for a median duration of 2 months (range, 0.5–8 months). The median duration of antimicrobial drug therapy after undergoing the last surgery to control the infection was 4 months (range, 1–19 months) (Table 2). There was no significant difference between different NTM strains in the total duration of therapy and the time from the last infection control surgery to cessation of antimicrobial therapy (Figure 1). The total duration of therapy and the time from the last infection control surgery to cessation of antimicrobial therapy was longer for immunosuppressed as compared to non-immunosuppressed patients (Figure 2).

## Discussion

Non-tuberculous mycobacterial species are highly resistant to common medical disinfectants, and the infections are mostly related to iatrogenic invasive procedures that are commonly considered in the treatment of traumatic fractures and in certain traumatic treatments (including puncture and acupuncture) (Cusumano et al., 2017; Dickison et al., 2019; Philips et al., 2019). In the series of cases investigated herein, the most common causes were identified to be previous history of musculoskeletal trauma, percutaneous inoculation, and prior bone and joint surgery, while only one patient exhibited the presence of NTM infection after undergoing prosthetic joint surgery, a cause that was different from the common causes of infection in prosthetic joint surgery reported in the literature (Buser et al., 2019; Goldstein et al., 2019). Among 25 patients, only three patients were immunosuppressed, a finding that was consistent with that published in previous reports (Park et al., 2014; Johnson and Stout, 2015). Notably, two immunosuppressed patients were infected with *M. abscessus* and *M. avium*, and both received treatment with a duration of over a year and a half. The hand/wrist has been reported to be the most frequent site of NTM infection (Park et al., 2014), while the most common site in our case series was the knee joint, and this might be explained by the fact that the patients were mostly farmers engaged in manual labour for many years.

The diagnostic value of routine laboratory tests for musculoskeletal NTM infection remains limited. We determined the presence of inflammatory markers, including ESR, C-reactive protein, and leucocyte counts. Whereas the ESR in our patients was found to be frequently elevated, the C-reactive protein and leucocyte counts were typically normal. Generally, imaging results do not aid in distinguishing between musculoskeletal infections caused by typical pyogenic bacteria and those caused by NTM species; however, musculoskeletal NTM infections are characterised by slow progression (Theodorou et al., 2001; Hogan et al., 2019). The imaging changes observed in the case series mainly comprised soft tissue swelling, joint effusion, osteopenia, marginal osseous erosions, and sinus tract, consistent with the changes reported in the literature (Theodorou et al., 2001). Histopathologically, acid-fast bacilli testing showed positive results in four patients with granulomatous inflammation and necrosis and in one patient with granulomatous inflammation and abscess. Notably, the pathological manifestations of infections with *M. tuberculosis* and NTM species are similar. Culture methods remain the gold standard for diagnosis of musculoskeletal NTM infection. As NTM species are ubiquitous in the environment, a single NTM isolate possesses the potential for causing contamination. Therefore, this study included positive cases for more than two tissue specimens or puncture specimens simultaneously. However, there are cases in which clinical parameters meet diagnostic criteria but no organisms are isolated. The reported prevalence of culture-negative infections ranges approximately from 10 to 30% (Rosteius et al., 2018). Metagenomic NGS is a sensitive diagnostic modality, particularly in culture-negative cases (Tsang et al., 2020). The small sample size of this study was a limitation; hence, it might be necessary to obtain additional data for comparison. It should be noted that although metagenomic NGS is an efficient approach, culture is deemed necessary for sensitivity testing owing to the differences observed in drug resistance traits among species.

The median treatment duration for all patients, except case 3, was 5 months, and this was shorter than the 13.5 months, 55 weeks, and 14 months reported in three previous case series (Park et al., 2014; Goldstein et al., 2019; Napaumpaiporn and Katchamart, 2019). This may be due to the fact that we considered traditional Chinese medicine in the treatment of musculoskeletal NTM infections, which involved an individualised treatment assisted by traditional Chinese medicine according to the physical conditions of each patient and a timely medication adjustment according to the respective physical recovery conditions. Except for one patient (case 3) who succumbed because of heart failure after debridement, the remaining 24 patients presented with complete cure without recurrence, for a complete recovery rate of 96% (24/25), compared with rates of 76% (22/29) and 93% (13/14) reported in two previous case series (Park et al., 2014; Goldstein et al., 2019). We emphasise the importance of prescriptive treatment to effectively reduce the generation of drug-resistant bacteria and to improve the cure rate. Eight patients were subjected to repeated debridement for infection control, and three patients were subjected to hardware removal to control the infection, including a patient (case 2) with a prosthetic joint. Additionally, as recommended by literature (Goldstein et al., 2019; Hogan et al., 2019), clinicians actively use antibiotic-eluting polymethyl methacrylate cement during surgery, delivering effective local antimycobacterial therapy. The agents that are active against NTM drugs and can be incorporated into cement are thermostable and include aminoglycosides, some cephalosporins, macrolides, carbapenems, and quinolones (Hogan et al., 2019).

Multidrug therapy includes the administration of intravenous and oral antibiotics based on antibiotic sensitivities. Owing to the toxicity and interactions of the drugs, 11 patients presented with adverse reactions, such as ototoxicity, gastrointestinal reactions, and hepatorenal toxicity. Therefore, at every stage, it is important to monitor routine blood parameters, liver and kidney functions, and other related indicators, and to conduct active management of cases in patients (Tang et al., 2020). Among the related detection indicators, ESR and CRP are used for the diagnosis of musculoskeletal NTM infection, while the white blood cell count is used for the diagnosis and monitoring of adverse reactions.

Of note, we comprehensively recorded the course of surgical treatment of patients in other hospitals. In 11 patients who underwent debridement in other hospitals, the infection was not controlled because the aetiology was negative, and NTM infection was not suspected. The long interval between the onset of symptoms in all patients and the identification of the source of infection and the conduction of subsequent treatment highlight the need for the early identification of NTM infections as well as improved laboratory testing capabilities and aggressive treatment.

Presently, factors such as the optimal time for treating musculoskeletal NTM infection, disease burden, degree of host immunosuppression, completeness of surgical debridement, clinical response, and other factors are not fully understood (Hogan et al., 2019). We retrospectively analysed cases of skeletal muscle infection with NTM species from two centres over a period of 5 years for obtaining complete case data to highlight: (1) In the diagnosis of musculoskeletal NTM infection, the combination of NGS and culture can identify the pathogen early and improve the diagnostic efficiency. (2) All patients underwent surgical intervention, and satisfactory results could be obtained by combining surgery with a specific anti-NTM drug therapy. (3) The treatment duration in immunosuppressed patients was longer than that in non-immunosuppressed patients. However, because the number of immunosuppressed patients in our study was very small, the results need to be interpreted with caution. More clinical data on immunosuppressed patients have to be collected to ensure the applicability of the results.

## Data Availability Statement

The raw data supporting the conclusions of this article will be made available by the authors, without undue reservation.

## Ethics Statement

Written informed consent was obtained from the individual(s) for the publication of any potentially identifiable images or data included in this article.

## Author Contributions

QM, RC, EY, YY, and NJ: acquisition of data, data analysis, manuscript preparation, study design, and laboratory work. YT, SW, BW, and WY: sample collection and critical review of the manuscript. QZ, BM, YH, and ZW: data analysis and critical review of the manuscript. YT and ZW: orthopaedic surgeons and analysed the cases. YY: critical review of the manuscript. YiL and YoL: design of the study and critical review of the manuscript. QM, EY, and YY: medical record collection and major contributors in draft preparation of the manuscript. All authors read and approved the final manuscript.

## Conflict of Interest

The authors declare that the research was conducted in the absence of any commercial or financial relationships that could be construed as a potential conflict of interest.

## Publisher’s Note

All claims expressed in this article are solely those of the authors and do not necessarily represent those of their affiliated organizations, or those of the publisher, the editors and the reviewers. Any product that may be evaluated in this article, or claim that may be made by its manufacturer, is not guaranteed or endorsed by the publisher.
