# Supplementary material for: Non-tuberculous Mycobacterial Infection of the Musculoskeletal System Detected at Two Tertiary Medical Centres in Henan, China, 2016–2020
Source: Front Microbiol. 2021 Dec 16;12:791918. doi: 10.3389/fmicb.2021.791918 (PMC8718100; doi:10.3389/fmicb.2021.791918)
Supplement: Supplementary file 1 [file Data_Sheet_1.doc]

Supplementary Material

# Supplementary Table 1. Antimicrobial susceptibility of mycobacteria species in patients with nontuberculous mycobacterial musculoskeletal infections, Henan, China*

| Case no. | Species | MIC value, µg/mL (susceptibility level) † | | | | | | | | | | | | | |
| --- | --- | --- | --- | --- | --- | --- | --- | --- | --- | --- | --- | --- | --- | --- | --- |
|  |  | AMK | AZM | FEP | FOX | CIP | CLR | DOX | IPM‡ | LZD | MIN | MXF | TGC | TOB | SXT |
|  |  |  |  |  |  |  |  |  |  |  |  |  |  |  |  |
| 1 | *M. smegmatis* |  |  |  |  |  |  |  |  |  |  |  |  |  |  |
| 2 | *M. abscessus* |  |  |  |  |  |  |  |  |  |  |  |  |  |  |
| 3 | *M. abscessus* |  |  |  |  | 1 (S) | 4(I) | >16 (R) | 16 (I) |  |  |  |  |  |  |
| 4 | *M. houstonense* |  | 16 | >32 | 32 (I) | 1 (S) | 2 (S) | >16 (R) |  |  |  |  |  |  |  |
| 5 | *M. abscessus* | <1 (S) |  | 2 | 32 (I) | <0.12 (S) | 1 (S) | 4 (I) | 4 | <1 (S) | >8(R) | >16 (R) |  | 8 (I) | >4/67 (R) |
| 6 | *M. fortuitum* | <1 (S) | 8 | >32 | 32 (I) | <0.12 (S) | 1 (S) | <0.12 (S) | <2 (S) | <1 (S) | <1 (S) | <0.25 (S) | 0.25 | <1 (S) | <0.25/4.8 (S) |
| 7 | *M. houstonense* | 4 (S) |  |  | 64 (I) | <0.12 (S) | 1 (S) | <0.12 (S) | <2 (S) | <1 (S) | <1 (S) | <0.25 (S) | <0.12 | 8 (I) | <0.25/4.8 (S) |
| 8 | *M. fortuitum* | 4 (S) |  | >32 | 64 (I) | 0.5 (S) | 1 (S) | 4 (I) | 16 (I) | <1 (S) | <1 (S) | <0.25 (S) |  | <1 (S) | <0.25/4.8 (S) |
| 9 | *M. fortuitum* | <1 (S) |  |  | 32 (I) | <0.12 (S) | 1 (S) | <0.12 (S) | >16 (R) | 2 (S) | <1 (S) | <0.25 (S) | 0.5 | <1 (S) | 0.5/9.5 (S) |
| 10 | *M. abscessus* | 8 (S) | 4 | >32 | 16 (S) | 0.5 (S) | 1 (S) |  |  |  |  |  |  |  | 0.5/9.5 (S) |
| 11 | *M. fortuitum* | 4 (S) |  | 2 | 16 (S) | 0.25 (S) | 1 (S) | <0.12 (S) |  |  | <1 (S) | >16 (R) | <0.12 | <1 (S) | <0.25/4.8 (S) |
| 12 | *M. abscessus* | 16 (S) | 4 |  | 16 (S) | 2 | 1 (S) | 0.5 (S) | <2 (S) | 2 (S) | >8(R) | >16 (R) |  |  | >4/67 (R) |
| 13 | *M. houstonense* | <1 (S) | 16 | >32 |  | 0.5 (S) | 4(I) |  | >16 (R) | <1 (S) | >8(R) | <0.25 (S) |  |  | 0.5/9.5 (S) |
| 14 | *M. fortuitum* | <1 (S) |  |  |  | 0.5 (S) | 2 (S) | 0.5 (S) |  | 2 (S) | <1 (S) |  | 1 | <1 (S) | <0.25/4.8 (S) |
| 15 | *M. avium* |  |  |  |  |  |  |  |  |  |  |  |  |  |  |
| 16 | *M. abscessus* |  | 8 | >32 | 16 (S) | 0.5 (S) | 0.5 (S) |  | <2 (S) |  | <1 (S) | >16 (R) | <0.12 | <1 (S) | >4/67 (R) |
| 17 | *M. abscessus* | 32 (I) | 8 |  |  | 0.25 (S) | 2 (S) | >16 (R) | 16 (I) | 16 (I) |  | >16 (R) | <0.12 | 8 (I) | 0.5/9.5 (S) |
| 18 | *M. abscessus* | <1 (S) | 4 | 2 | 16 (S) | <0.12 (S) | 2 (S) | 4 (I) | <2 (S) | <1 (S) | >8(R) | >16 (R) | 0.25 | >16 (R) | >4/67 (R) |
| 19 | *M. fortuitum* | <1 (S) |  | >32 |  | 1 (S) | 2 (S) |  | <2 (S) | <1 (S) |  | <0.25 (S) | <0.12 | <1 (S) | 0.5/9.5 (S) |
| 20 | *M. fortuitum* | <1 (S) | 16 |  |  | 1 (S) | 2 (S) | 0.5 (S) | 8(I) | 2 (S) | >8(R) |  | <0.12 | >16 (R) | <0.25/4.8 (S) |
| 21 | *M. fortuitum* | <1 (S) | 8 | >32 |  | 1 (S) | 1 (S) | 0.5 (S) | <2 (S) | 4 (S) |  | <0.25 (S) | <0.12 | <1 (S) | <0.25/4.8 (S) |
| 22 | *M. avium* |  |  |  |  |  |  |  |  |  |  |  |  |  |  |
| 23 | *M. fortuitum* | <1 (S) | 8 | >32 | 16 (S) | 1 (S) | 1 (S) |  |  | <1 (S) | <1 (S) | <0.25 (S) | 0.25 | <1 (S) | <0.25/4.8 (S) |
| 24 | *M. fortuitum* | <1 (S) | 8 | 2 | 16 (S) | 1 (S) | 1 (S) | >16 (R) | <2 (S) | <1 (S) |  | <0.25 (S) | 0.25 | 8 (I) | <0.25/4.8 (S) |
| 25 | *M. fortuitum* | 8 (S) |  | >32 | 64 | <1 (S) | 2 (S) | 2 (I) | <2 (S) | 2 (S) | >8 (R) | <0.5 (S) | <0.12 | 4 (S) | <0.5/9.5 (S) |

*Susceptibility levels are S, susceptible; I, intermediate; and R, resistant. If unspecified, Clinical Laboratory Standards Institute (CLSI) guidelines do not clearly establish susceptibility breakpoints for these agents.

†Antimicrobial drugs tested used were AMK, amikacin; AZM, azithromycin; CIP, ciprofloxacin; CLR, clarithromycin; DOX, doxycycline; FEP, cefepime; FOX, cefoxitin; GEN, gentamycin; IPM, imipenem; LZD, linezolid; MXF, moxifloxacin; RIF, rifampin; SXT, trimethoprim-sulfamethoxazole; TGC, tigecycline; TOB, tobramycin.

‡Imipenem breakpoints are tentative and should be interpreted as such, pending further information.
